# Improved compressed sensing and super‐resolution of cardiac diffusion MRI with structure‐guided total variation

**DOI:** 10.1002/mrm.28245

**Published:** 2020-03-03

**Authors:** Irvin Teh, Darryl McClymont, Eric Carruth, Jeffrey Omens, Andrew McCulloch, Jürgen E. Schneider

**Affiliations:** ^1^ Leeds Institute of Cardiovascular & Metabolic Medicine University of Leeds Leeds United Kingdom; ^2^ Perspectum Diagnostics Ltd Oxford United Kingdom; ^3^ Geisinger Health System Danville Pennsylvania; ^4^ Department of Medicine University of California San Diego La Jolla California; ^5^ Department of Bioengineering University of California San Diego La Jolla California

**Keywords:** cardiac MRI, compressed sensing, diffusion kurtosis, diffusion tensor imaging, directional total variation, super‐resolution

## Abstract

**Purpose:**

Structure‐guided total variation is a recently introduced prior that allows reconstruction of images using knowledge of the location and orientation of edges in a reference image. In this work, we demonstrate the advantages of a variant of structure‐guided total variation known as directional total variation (DTV), over traditional total variation (TV), in the context of compressed‐sensing reconstruction and super‐resolution.

**Methods:**

We compared TV and DTV in retrospectively undersampled ex vivo diffusion tensor imaging and diffusion spectrum imaging data from healthy, sham, and hypertrophic rat hearts.

**Results:**

In compressed sensing at an undersampling factor of 8, the RMS error of mean diffusivity and fractional anisotropy relative to the fully sampled ground truth were 44% and 20% lower in DTV compared with TV. In super‐resolution, these values were 29% and 14%, respectively. Similarly, we observed improvements in helix angle, transverse angle, sheetlet elevation, and sheetlet azimuth. The RMS error of the diffusion kurtosis in the undersampled data relative to the ground truth was uniformly lower (22% on average) with DTV compared to TV.

**Conclusion:**

Acquiring one fully sampled non‐diffusion‐weighted image and 10 diffusion‐weighted images at 8× undersampling would result in an 80% net reduction in data needed. We demonstrate efficacy of the DTV algorithm over TV in reducing data sampling requirements, which can be translated into higher apparent resolution and potentially shorter scan times. This method would be equally applicable in diffusion MRI applications outside the heart.

## 1 INTRODUCTION

Diffusion tensor imaging is a method for noninvasive assessment of tissue microstructure, and measures water diffusion that serves as a surrogate marker of features such as cell integrity and tissue anisotropy.^1^ Diffusion tensor imaging is used in a wide range of applications and diseases, such as the assessment of neurodegenerative disorders,^2^ chronic kidney disease,^3^ and prostate cancer.^4^ In the heart, for example, diffusion tensor imaging (DTI) has been used to identify changes in myocardial sheetlet dynamics in patients with hypertrophic cardiomyopathy and dilated cardiomyopathy,^5^, ^6^, ^7^ and to assess remodeling in the heart following infarction.^8^ However, DTI is inherently slow, due to the need to acquire at least seven images, including images that are diffusion‐weighted (DW) in at least six unique directions, and one non‐DW image. For example, for robust measurements, in the presence of noise, 20 or more unique diffusion directions are recommended, thereby extending scan times.^9^, ^10^ In cardiac imaging,^11^ scan times are further prolonged by the need to acquire multiple averages to compensate for motion and short T_2_.

To address this limitation, several methods have been proposed that can accelerate DTI and diffusion MRI in general, including parallel imaging,^12^ simultaneous multislice imaging,^13^, ^14^, ^15^ and compressed sensing.^16^ The former two methods typically operate in image space or k‐space, and rely on dedicated multichannel RF coils, and their performance depends on the spatially variant noise amplification characterized by the geometry or *g*‐factor of the coil. Given that diffusion MRI requires multiple acquisitions with different diffusion‐encoding directions and magnitudes of diffusion weighting in *q*‐space, compressed‐sensing approaches are well‐placed to additionally exploit redundancies in *q*‐space. Applications of compressed sensing include reconstruction of complex fiber architecture in the brain from single *q*‐shell data,^17^ and recovery of fiber orientation information in the presence of 3‐8× undersampling of data.^18^, ^19^ Joint k‐*q*‐space acceleration has been proposed, including the use of ℓ_1_ regularization and motion compensation,^20^, ^21^ and phase‐constrained low‐rank models.^22^ These used spiral or pseudo‐randomized undersampling in both k‐space and *q*‐space to achieve 4‐fold time savings and/or reductions in image distortion. Furthermore, k‐*b* principal component analysis has been proposed for the reconstruction of up to 6× undersampled intravoxel‐incoherent motion imaging data in human brain.^23^

Structural total variation is a recently introduced regularizer that allows the reconstruction of images using edge information from a reference image.^24^ This approach, and in particular the variant of it known as directional total variation (DTV), in which both the location and orientation of edges is used, has been demonstrated to great effect in phantoms as well as T_1_‐weighted and T_2_‐weighted MRI in the brain. One advantage of this algorithm, in particular over the method of Jiang and Hsu,^25^ is that the contrast of the reference image has no effect on the image to be reconstructed. This makes it an ideal candidate for DWI reconstruction, in which images with similar image structure, but different diffusion contrast, are acquired. In particular, non‐DW images are well‐suited to being used as a reference image for DWI reconstruction, as the SNR is typically higher.

Although acceleration of image acquisition is one relevant aspect, undersampling of k‐space or *q*‐space is only relevant in acquisitions involving multiple shots or diffusion MRI data with multiple, and typically large, numbers of q‐samples. Where images are acquired in a single shot, as is often the case in single‐shot EPI diffusion MRI, data undersampling may be used in conjunction with super‐resolution techniques to instead improve image resolution and minimize image distortions. Various algorithms have been proposed to improve the resolution of diffusion MRI. Peled and Yeshurun applied a method based on subvoxel shifts^26^; however, the viability of this method for improving resolution has been questioned,^27^ as a subvoxel shift is equivalent to a linear phase offset in k‐space. Jiang and Hsu used reduced encoding imaging^28^ to modify the edges of k‐space from a high‐resolution reference image.^25^ Poot proposed a method that combines information from thick 2D slices acquired in a unique orientation for each DW image.^29^ Alexander et al proposed a method based on image‐quality transfer to perform super‐resolution in diffusion MRI.^30^ As with all methods based on machine learning, this approach relies on the training data being representative of any images that are being reconstructed.

In this paper, we demonstrate the suitability of the DTV regularizer for the reconstruction of diffusion‐weighted images. This is demonstrated by retrospectively undersampling and reconstructing images using both a compressed‐sensing framework with random undersampling of k‐space, and in a super‐resolution framework that reconstructs down‐sampled images through k‐space truncation. The source data consist of ex vivo rat hearts imaged at high resolution on a preclinical MR scanner, and the reconstructions are validated against fully sampled MR images. Although the algorithm is performed on ex vivo cardiac data, it is equally applicable to DW images of other organs, in which there is structural similarity between a reference image and DW images.

## 2 THEORY

We consider the process of image formation to be formulated as an inverse problem. Let 3D volume **Z** be sampled under the process **A** to yield discrete data **X**. Assuming additive Gaussian noise ε, this is modeled as X=AZ+ε. The process **A** can include a combination of spatial transforms, integral transforms (such as the Fourier transform in MRI, or the Radon transform in CT), convolution with a point spread function, and data sampling. Assuming that the problem is well posed, the reconstructed image **Y** may be obtained as follows:

(1) $$Y^{*}=\underset{Y}{\text{argmin}}{\left(\mathrm{AY}\;-\;X\right)}_{2}^{2}$$

In many cases, such as in super‐resolution or compressed sensing, this problem is badly conditioned or underdetermined. A common approach to addressing this is in the use of a regularizer, in the form of

(2) $$Y^{*}=\underset{Y}{\text{argmin}}{\left(\mathrm{AY}\;-\;X\right)}_{2}^{2}+\lambda R\left(Y\right).$$

The regularizer, *R*, introduces an assumption to the solution, such as controlling the smoothness of the image. Common choices for regularizers include TV^31^ or squared Laplacian.^29^ We consider the DTV method of Ehrhardt et al.^24^

### 2.1 Directional total variation

The TV of a 3D image **A** may be expressed as

(3) $$\text{TV}\left(A\right)=\sum_{n=1}^{3}\left|\nabla A_{n}\right|.$$

The 3D DTV constraint *J* applied to image **A** with reference image **B** is defined as^24^

(4) $$J\left(A,\;B\right)=\sum_{n=1}^{3}\left|D_{n}\nabla A_{n}\right|,$$

where matrix field $D_{n}\in M^{3}=I-\xi_{n}\xi_{n}^{*}$; I is the identity matrix; and $\xi_{n}:=\;\frac{\nabla B_{n}}{{\left|\nabla B_{n}\right|}_{\eta }}$. Tuning parameter η relates to the size of the edges in reference image **B**.

Images are reconstructed by minimizing the 3D DTV while maintaining consistency with the acquired data, as follows:

(5) $$Y^{*}=\;\underset{Y}{\text{argmin}}{∥\mathrm{FY}\;-\;X∥}_{2}^{2}\;+\;\lambda J\left(Y,\;I_{\text{ref}}\right)$$

where **Y** is the reconstructed image; **X** is the sampled data; **F** consists of the 3D Fourier transform and the sampling operator; and $I_{\text{ref}}$ is the reference image with the same size as **Y**.

Two experiments were performed to demonstrate the algorithm. The first applies the DTV before the problem of reconstructing randomly undersampled data (ie, a compressed‐sensing‐style acquisition). The second applied the prior to the problem of super‐resolution. In both experiments, Equation 5 was solved using the algorithm and software described previously.^24^ As the original software operated on magnitude‐only 2D images, modifications were made to extend it to operate on complex 3D images. The modified software will be made available upon request.

## 3 METHODS

### 3.1 Data acquisition

Ex vivo rat heart data were acquired in a previous study.^32^ Briefly, hearts were excised from Sprague Dawley rats (*N* = 5). Isolated hearts were perfused in Langendorff constant pressure mode with modified Krebs‐Henseleit solution, cardioplegically arrested with high potassium, and then perfused and stored in low‐osmolality Karnovsky's fixative doped with 2 mM gadolinium solution (ProHance; Bracco, Minnesota). Before imaging, the fixed hearts were washed in phosphate‐buffered saline + 2 mM gadolinium, and embedded in 1% agarose gel (Web Scientific, Crewe, United Kingdom). Nonselective 3D fast spin‐echo DTI data were acquired on a 9.4T preclinical MRI scanner (Agilent, Santa Clara, CA) with transmit/receive birdcage coil (inner diameter = 20 mm; Rapid Biomedical, Rimpar, Germany). Acquisition parameters were as follows: TR = 250 ms, TE = 9.3 ms, echo spacing = 4.9 ms, echo train length = 8, FOV = 20 × 16 × 16 mm, resolution = 100 μm^3^ isotropic, *b* = 1000 s/mm^2^, diffusion duration (δ) = 2 ms, diffusion time (Δ) = 5.5 ms, and number of DW directions = 10. Three‐dimensional spoiled gradient‐echo anatomical images were acquired with the same FOV, TR = 20 ms, TE = 4 ms, flip angle = 30°, and resolution = 33 μm^3^ isotropic. Noise data were acquired using an identical sequence without RF pulses and with TR minimized. The shims, receiver gain, and center frequency were unchanged during acquisition of the noise data. Experimental investigations conformed to the UK Home Office guidance on the Operations of Animals (Scientific Procedures) Act 1986 and were approved by the University of Oxford ethical review board.

To demonstrate application in cardiomyopathy, we examined data from a previous study^33^ comparing male Sprague Dawley rats with sham surgery (*N* = 4) and transverse aortic constriction (TAC; *N* = 4). Use of these rats followed National Institutes of Health guidelines and were approved by the University of California San Diego Institutional Animal Care and Use Committee. Hearts were excised, arrested, fixed, and embedded for imaging in the same manner as described previously. Diffusion spectrum imaging data were acquired using a fast spin‐echo sequence: TR = 250 ms, TE = 15 ms, echo spacing = 4 ms, echo train length = 8, FOV = 21.6 × 14.4 × 14.4 mm, resolution = 180 μm^3^ isotropic, *b*_max_ = 10 000 s/mm^2^, and number of DW directions = 514. For further details on the generation of the TAC model and pulse sequence, please refer to McClymont et al.^33^

### 3.2 Experiment 1: Random undersampling in k‐space

In experiment 1, the data were randomly retrospectively undersampled in k‐space. A random sampling mask was applied based on a Gaussian distribution of samples in the two phase‐encoding directions. The central region of k‐space was fully sampled, using a circle with a radius of 10 samples. The readout dimension was fully sampled. The acceleration factor for the 10 DW images, $A_{\mathrm{dw}}$, varied between 2 and 10. The non‐DW image was fully sampled and used as the reference image. Therefore, the effective acceleration for the acquisition, $A_{\mathrm{eff}}$, was

(6) $$A_{\mathrm{eff}}=\;\frac{11}{1+\frac{10}{A_{\mathrm{dw}}}}.$$

The undersampled data were reconstructed using both traditional TV (Equation 3) and DTV (Equation 4) as priors. The optimization settings for the DTV and TV reconstruction algorithms were identical. The algorithm was run with 200 iterations, and tuning parameter λ was set such that ${\left\|\mathrm{FY}\;-\;X\right\|}_{2}^{2}\;\approx \;\sigma^{2}$, where $\sigma^{2}$ is the measured variance of the noise. The noise level was computed based on the real component of the noise data in k‐space according to our previous work.^16^ In the DTV reconstruction, η was set to 0.05. The value of *η* was empirically determined as a reasonable compromise between allowing edge information to be reconstructed in the target image without introducing spurious edges.

### 3.3 Experiment 2: Super‐resolution using non‐DW reference

**Figure 1:**
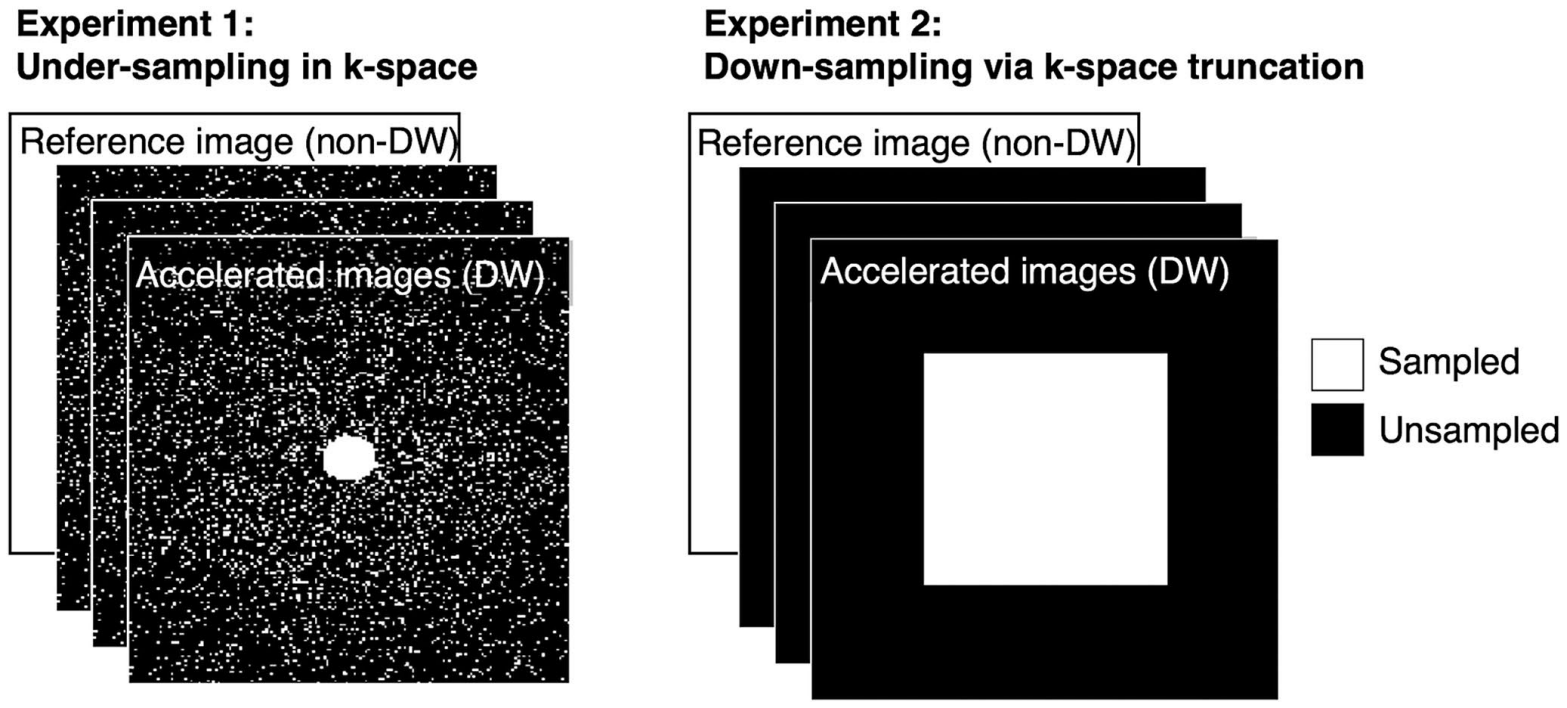
Masks for retrospective undersampling of k‐space. In Experiment 1, masks in 2D were generated based on random sampling with a Gaussian distribution along the first and second phase‐encoding directions (in‐plane). The central region and the readout dimension (through‐plane) were fully sampled. The number of remaining k‐samples was equal to the total number of samples in 3D divided by the acceleration factor, $A_{\mathrm{dw}}$. In Experiment 2, the masks retained only the central region of k‐space, where the number of samples in each dimension were down‐sampled by a factor of $A_{\mathrm{dw}}$

In experiment 2, the central portion of k‐space was selected to simulate a low‐resolution acquisition, effectively down‐sampling the image as shown in Figure 1. As in experiment 1, the non‐DW image was used as a reference, and was not down‐sampled. The DW images were down‐sampled in each dimension by a factor of 2, 4, 6, 8, and 10, effectively reducing the isotropic resolution to 200 μm, 400 μm, 600 μm, 800 μm, and 1 mm, respectively. The retrospective undersampling masks for experiments 1 and 2 are shown (Figure 1).

In both experiments 1 and 2, the reconstructed images were validated using the original fully sampled data. The magnitude of the images was fit to a diffusion tensor model, and the mean diffusivity and fractional anisotropy were calculated. The helix angle, transverse angle, sheetlet elevation, and sheetlet azimuth were generated using the local coordinate system described.^32^ The RMS error (RMSE) in the myocardium of these six parameters was calculated, and the mean and SD of the RMSE across the five hearts is reported. In addition, the DTV algorithm was applied using the high‐resolution anatomical data as a reference.

In the sham and TAC groups, the DW data were down‐sampled by a factor of 8 using both random undersampling and super‐resolution approaches described in Experiments 1 and 2. We showed previously that the diffusion kurtosis was useful in discriminating the sham versus TAC groups where DTI was not. The beta distribution model was therefore fitted to the diffusion spectrum imaging data, and the diffusion kurtosis was obtained along the principal eigenvectors of the diffusion tensor.^33^ The kurtosis and RMSE of the kurtosis relative to the fully sampled case were reported in the left ventricular myocardium in a single long‐axis slice. We evaluated whether there were significant differences in kurtosis between sham and TAC hearts, and in the RMSE of the kurtosis between TV and DTV reconstructions. Unpaired two‐tailed t‐tests were performed, and *P* = .05 significance level was used.

## 4 RESULTS

**Figure 2:**
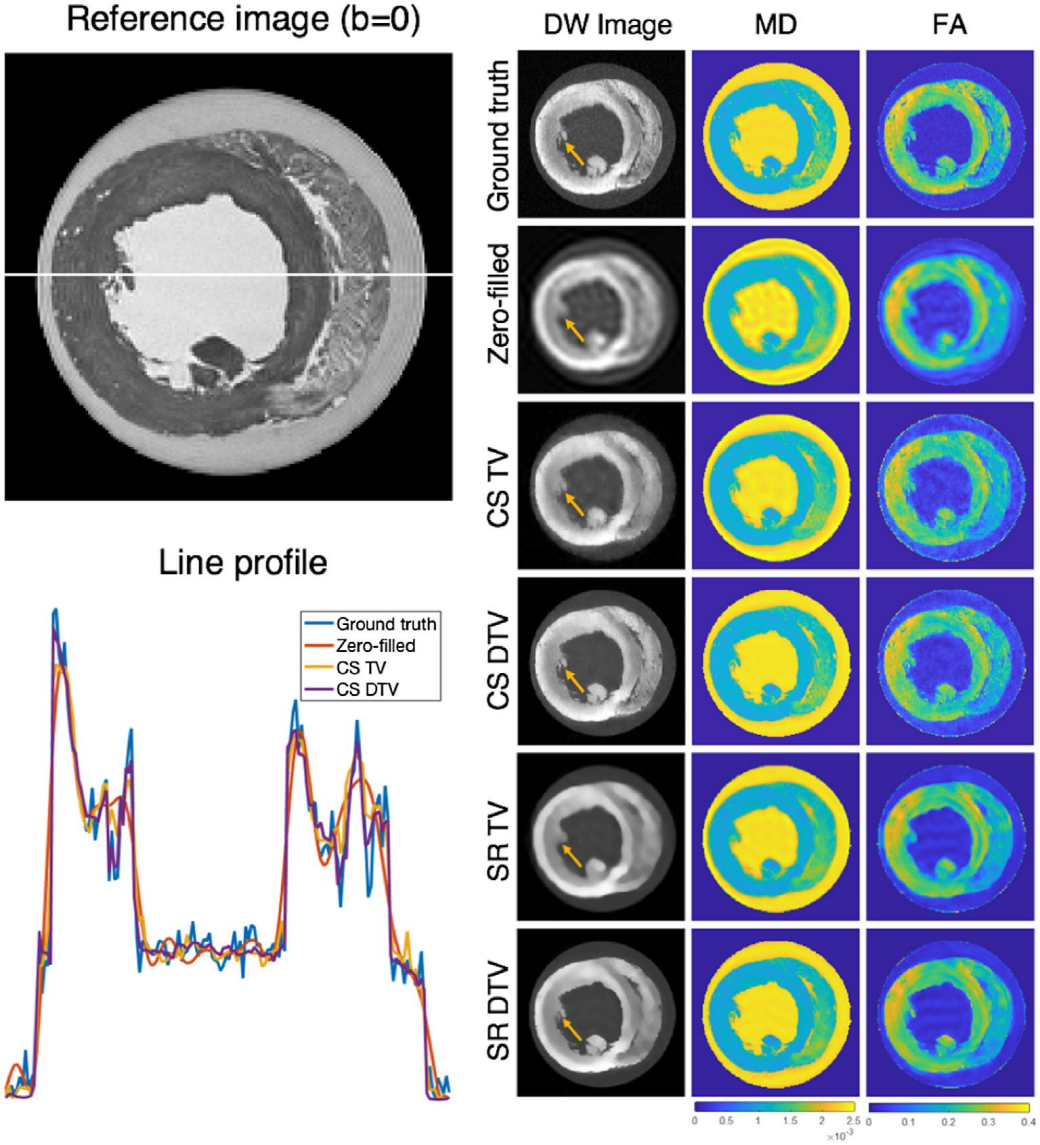
(Right) Representative diffusion‐weighted (DW) images, mean diffusivity (MD; mm^2^/s), and fractional anisotropy (FA) maps in a midventricular slice. (Top to bottom) Fully sampled ground truth, zero‐filled, compressed‐sensing (CS) total variation (TV) and directional total variation (DTV), and super‐resolution (SR) TV and DTV data, where CS data were undersampled by 6× and the zero‐filled and SR data were undersampled by 6^3^. Ground‐truth and zero‐filled data are shown for comparison. Improvements in edge definition are shown in data reconstructed with DTV (arrows). (Top left) A reference non‐DW image is shown, and (bottom left) corresponding line profiles through the lateral and septal walls in the DW images show better fidelity compared with the ground‐truth data when using DTV. The SR line profiles are not shown for clarity

**Figure 3:**
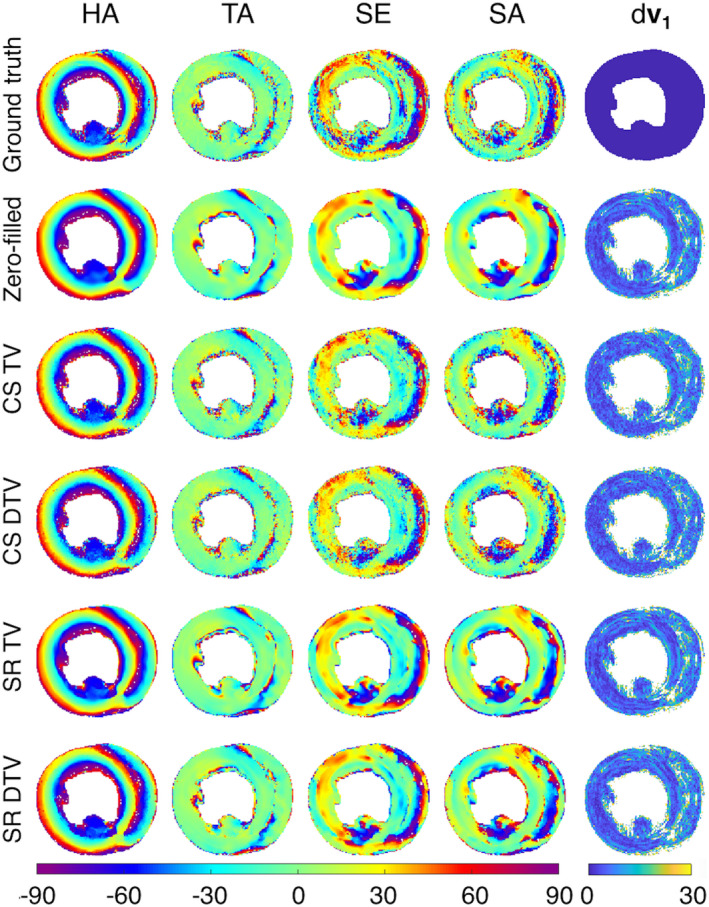
(Left to right) Representative helix angle (HA), transverse angle (TA), sheetlet elevation (SE), sheetlet azimuth (SA) maps, and the angle between reconstructed and ground‐truth primary eigenvectors (d**v**_1_) (in °). (Top to bottom) Fully sampled ground truth, zero‐filled, CS TV and CS DTV, and SR TV and SR DTV data, where CS data were undersampled by 6× and the zero‐filled and SR data were undersampled by 6^3^

Figure 2 presents sample parameter maps for the reconstruction algorithms. Heart 5, with the DW images down‐sampled to 600 μm, was selected as representative of the algorithm performance. Zero‐filled images show significant blurring and Gibbs ringing. In the super‐resolution reconstructions, the traditional TV algorithm effectively removes Gibbs ringing, but does not sharpen edges. In comparison, the DTV algorithm removes Gibbs ringing and sharpens the image at the interfaces between tissue and gel/buffer. Edges that were not present in the low‐resolution image (such as the observed fine structures within the right ventricle) were not introduced in the TV or DTV reconstructed image. In the case of compressed sensing, we present data undersampled by 6×. Here, the observed fine structures were better preserved compared with the super‐resolution data, and edges were sharper using DTV compared with TV. Figure 3 shows that helix angle and transverse angle in all cases are close to the ground truth due to their inherent smoothness. However, the blurring in the zero‐filled and super‐resolution data exceeds that of the data reconstructed with compressed sensing. This is reflected in larger angles between the reconstructed and ground‐truth primary eigenvectors.

**Figure 4:**
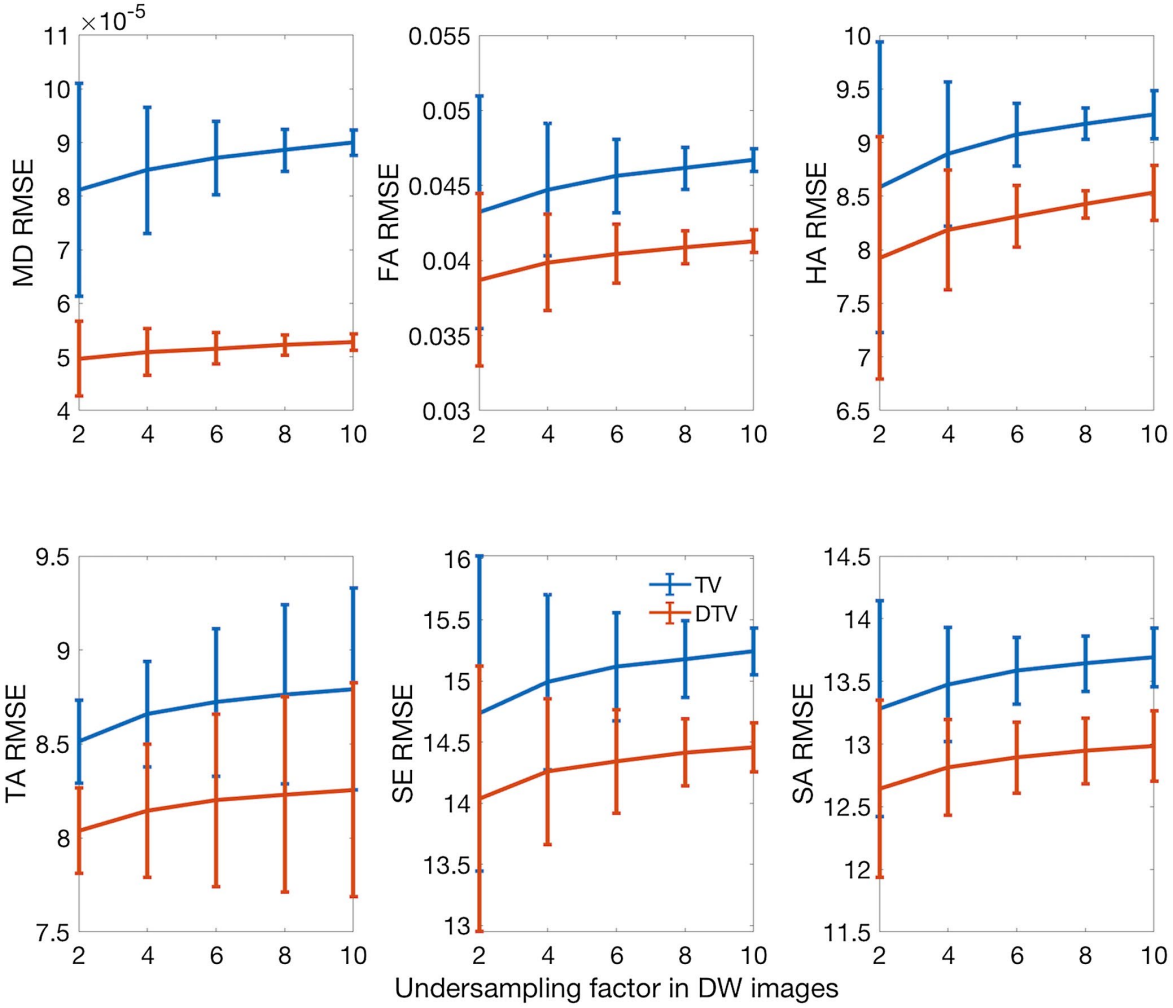
Root mean square error (RMSE) from experiment 1 with random undersampling of k‐space shows greater agreement of DTV reconstructions with the fully sampled ground‐truth data, compared with that of the TV reconstructions. Mean diffusivity (mm^2^/s), FA, HA (º), TA (º), SE (º), and SA (º) are reported (mean ± SD across hearts)

The results of experiment 1 representing the compressed‐sensing approach are presented in Figure 4. The data show that the RMSE in the DTV reconstructions were uniformly lower than that of TV, as measured against the ground‐truth data. At an undersampling factor of 8, for example, the RMSE of the TV/DTV reconstructions were mean diffusivity ([8.86 ± 0.39] × 10^−5^/[5.22 ± 0.19] × 10^−5^ mm^2^/s), fractional anisotropy (0.0461 ± 0.0014/0.0409 ± 0.0011), helix angle (9.18º ± 0.15º/8.42º ± 0.13º), transverse angle (8.76º ± 0.48º/8.23º ± 0.52º), sheetlet elevation (15.18º ± 0.31º/14.42º ± 0.27º), and sheetlet azimuth (13.64º ± 0.22º/12.94º ± 0.26º), respectively.

**Figure 5:**
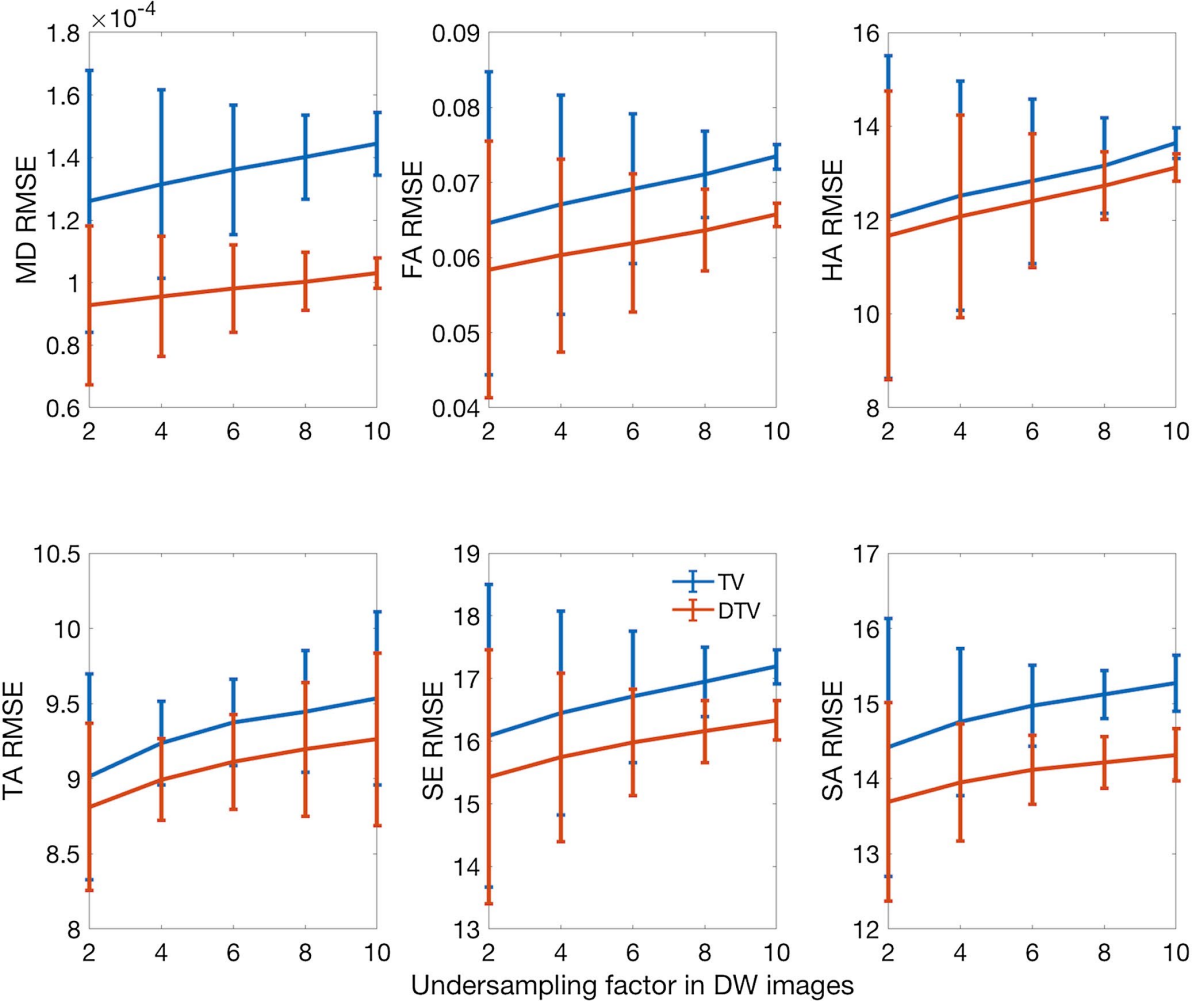
Root mean square error from experiment 2 with down‐sampling of k‐space by 2 ≤ $A_{\mathrm{dw}}$ ≤ 10 in each dimension, resulting in effective undersampling of $A_{\mathrm{dw}}$ ^3^, and reducing the effective isotropic resolution to 200, 400, 600, 800, and 1000 μm, respectively. Directional TV yielded consistently lower RMSE than TV reconstructions. Mean diffusivity (mm^2^/s), FA, HA (º), TA (º), SE (º), and SA (º) are reported (mean ± SD across hearts)

The results of experiment 2 indicate that the DTV algorithm consistently yields lower RMSE than the TV algorithm (Figure 5). At an undersampling factor of 8, corresponding to an effective isotropic resolution of 800 μm, the RMSE of the TV/DTV reconstructions were MD (1.40 ± 0.14] × 10^−4^/[1.00 ± 0.09] × 10^−4^ mm^2^/s), fractional anisotropy (0.0711 ± 0.0058/0.0637 ± 0.0055), helix angle (13.17º ± 1.02º/12.73º ± 0.73º), transverse angle (9.45º ± 0.41º/9.20º ± 0.45º), sheetlet elevation (16.95º ± 0.56º/16.16º ± 0.49º), and sheetlet azimuth (15.12º ± 0.32º/14.21º ± 0.35º), respectively.

**Figure 6:**
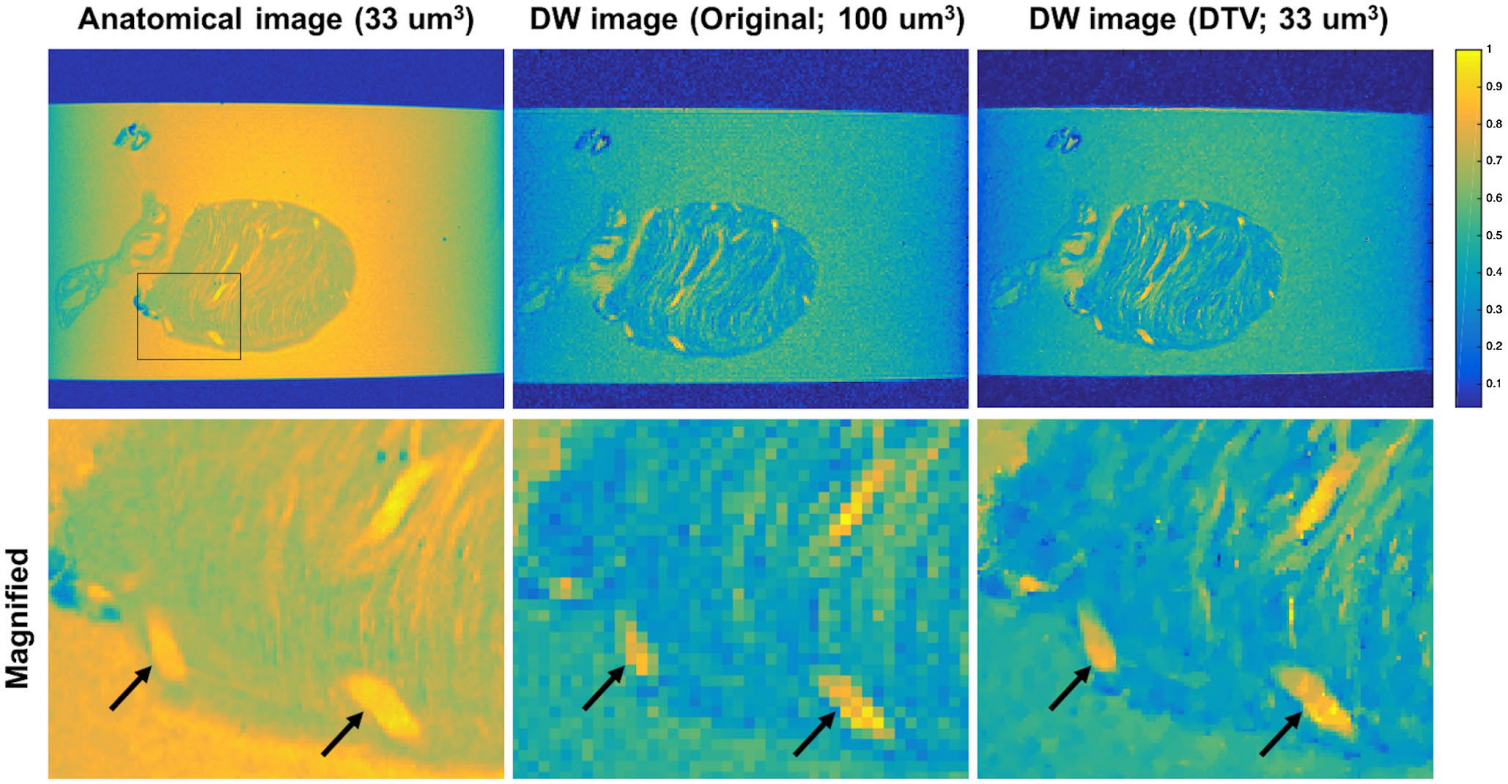
Representative DW image reconstructed at 33 μm^3^ isotropic resolution using the DTV algorithm and the high‐resolution anatomical data as a reference. The DTV reconstructed image is less pixelated than the original image, with structures such as large vessels (arrows) corresponding well with the anatomical image

The DW images reconstructed to 33 μm^3^ isotropic resolution using the DTV algorithm and the high‐resolution anatomical data as a reference are shown in Figure 6.

**Figure 7:**
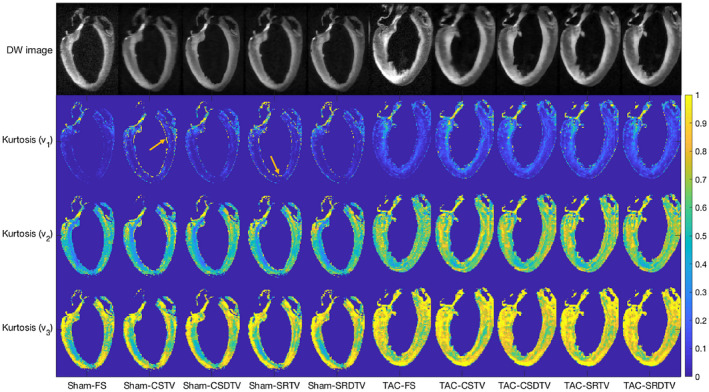
Diffusion‐weighted images and kurtosis maps in representative sham and transverse aortic constriction (TAC) hearts. (Top to bottom) Single DW image at *b* = 2012 s/mm^2^ and diffusion kurtosis along principal eigenvectors of the diffusion tensor **v_1_**,** v_2_**, and **v_3_**. (Left to right) Sham heart data reconstructed with fully sampled (FS) k‐space in the DW images, and 12.5% k‐space in the DW images reconstructed with CS, SR, TV, and DTV. Artifactually high kurtosis was seen along **v_1_** in the subendocardium of the TV‐reconstructed sham heart data (arrows)

Figure 7 depicts the sample DW images at *b* = 2012 s/mm^2^ and the kurtosis along principal eigenvectors of the diffusion tensor **v_1_**,** v_2_**, and **v_3_** as reconstructed using compressed‐sensing, super‐resolution, TV, and DTV methods. Fully sampled data are shown as a reference. Edges are generally better preserved in the DTV reconstructed data, and regions of artifactually high kurtosis can be seen particularly in the TV reconstructed sham heart data.

**Figure 8:**
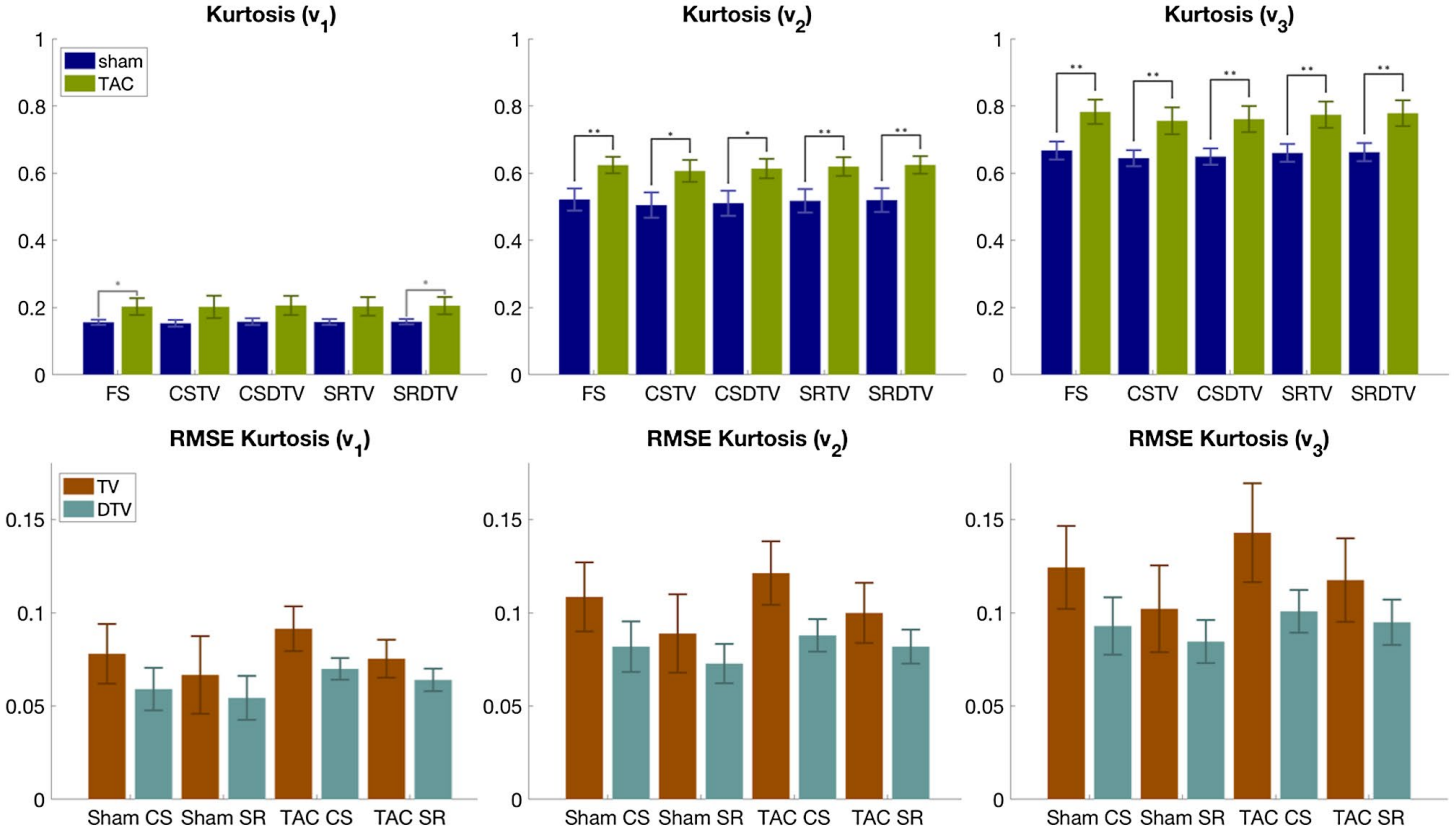
(Top) Diffusion kurtosis along principal eigenvectors of the diffusion tensor **v_1_**,** v_2_**, and **v_3_** (mean ± SD across hearts). Unpaired two‐tailed t‐tests were performed between corresponding sham‐TAC pairs with *P* < .05 (*) and *P* < .01 (**). Data reconstructed with FS, CS, SR, TV, and DTV approaches. (Bottom) Corresponding RMSE of diffusion kurtosis with respect to the fully sampled data (mean ± SD across hearts). The differences between TV and DTV pairs were not significant at *P* = .05

Figure 8 and Table 1 show that the kurtosis is higher in the TAC hearts compared with the sham hearts, regardless of the method of undersampling and reconstruction. The differences were significant in all cases of kurtosis along **v_2_** and **v_3_**. Furthermore, only the super‐resolution DTV reconstructed data showed significant differences in the kurtosis along **v_1_** between TAC and sham, which were present in the fully sampled data. Artifactually high kurtosis along **v_1_** was observed in the TV reconstructed data, particularly along the subendocardium of the sham heart. Across sham, TAC, compressed sensing, and super‐resolution groups, the RMSE of the kurtosis was consistently lower, by 22% on average, in the DTV compared with the TV reconstructed data, although the differences were not significant.

**Table 1:** Diffusion kurtosis and its RMSE along principal eigenvectors of the diffusion tensor **v_1_**,** v_2_**, and **v_3_** as illustrated in Figure 8 (mean ± SD across hearts)

			v_1_			v_2_			v_3_		
			Mean	SD	*P*	Mean	SD	*P*	Mean	SD	*P*
Kurtosis	FS	Sham	0.156	0.008	.043	0.522	0.033	.006	0.667	0.027	.005
		TAC	0.203	0.025		0.624	0.025		0.783	0.036	
	CS TV	Sham	0.153	0.010	.081	0.505	0.038	.013	0.645	0.024	.010
		TAC	0.202	0.033		0.607	0.033		0.756	0.040	
	CS DTV	Sham	0.158	0.010	.054	0.510	0.037	.010	0.649	0.024	.008
		TAC	0.206	0.029		0.614	0.029		0.761	0.039	
	SR TV	Sham	0.157	0.009	.057	0.518	0.035	.008	0.660	0.026	.008
		TAC	0.203	0.028		0.620	0.028		0.774	0.039	
	SR DTV	Sham	0.158	0.008	.044	0.520	0.035	.007	0.663	0.027	.007
		TAC	0.206	0.026		0.625	0.026		0.779	0.038	
RMSE Kurtosis	Sham CS	TV	0.078	0.016	.291	0.109	0.018	.414	0.124	0.022	.389
		DTV	0.059	0.011		0.082	0.014		0.093	0.015	
	Sham SR	TV	0.067	0.021	.209	0.089	0.021	.545	0.102	0.023	.504
		DTV	0.054	0.012		0.073	0.011		0.085	0.012	
	TAC CS	TV	0.091	0.012	.548	0.121	0.017	.500	0.143	0.026	.441
		DTV	0.070	0.006		0.088	0.009		0.101	0.011	
	TAC SR	TV	0.075	0.010	.269	0.100	0.016	.303	0.117	0.022	.327
		DTV	0.064	0.006		0.082	0.009		0.095	0.012	

Note

*P*‐values of corresponding sham‐TAC pairs and TV‐DTV pairs are reported.

## 5 DISCUSSION

In this work, the DTV algorithm of Ehrhardt et al was applied to diffusion MRI, with the aim of accelerating the acquisition with minimal loss in image quality and accuracy of diffusion metrics. An 8× undersampling in the DW data resulted in an 80% net reduction in data requirements, factoring in fully sampled non‐DW data, which could be translated into time savings.

Image reconstruction was achieved by leveraging edge information provided by a reference image to build the peripheral portions of k‐space. The DTV algorithm works by promoting edges in reconstructed images occurring in regions where edges are known to exist in the reference image. This is in contrast to the original TV algorithm, which penalizes all edges. Therefore, enough data in the center of k‐space must be acquired to drive the image contrast.

Because each DW image is reconstructed individually, the reconstruction algorithms presented here do not impose any specific model on the data. This also means that the reconstruction is highly parallelizable. However, it is possible to additionally impose a model that uses the DWI relationships in *q*‐space, such as the diffusion tensor model or compartmental model,^34^ to further constrain the reconstructed image.

The random acquisition scheme used in experiment 1 yielded better results than the truncated k‐space scheme used in experiment 2, due to the incoherence of the random undersampling pattern with TV. In experiment 2, the reconstruction algorithm was effective at removing ringing and sharpening large edges, but did not re‐introduce the fine details that were lost in the down‐sampling process. Among the parameters reported, the difference in RMSE between TV and DTV reconstructions was greatest in the mean diffusivity. This is due to the DTV algorithm more efficiently reducing blurring than the TV algorithm. Consequently, the effect of partial volume at the interfaces of the myocardium with the gel and buffer, wherein the latter have considerably higher diffusivities than the myocardium, is mitigated with DTV. Across parameters, there appears to be a general trend toward increasing RMSE with decreasing SD as the undersampling factor is increased. The increasing RMSE can be explained by the increased smoothing of the images at higher undersampling. The decreasing SD of RMSE across the hearts suggests that the RMSE plateaus at higher undersampling.

The results in sham and TAC hearts showed that DTV consistently outperformed TV in terms of RMSE of the kurtosis relative to the fully sampled data. We observed that the kurtosis was consistently higher in TAC than sham hearts, particularly in the directions of the second and third diffusion eigenvectors, recapitulating our previous observations in fully sampled data.^33^ We additionally show that DTV was able to detect significant differences in kurtosis along **v_1_** between TAC and sham hearts, where TV was unable to. We note that the sample size was small, and similar trends, not reaching statistical significance, were observed in the TV data. The artifactually high kurtosis at the subendocardial border with the buffer in the TV reconstructed data are reflective of the contributions of TV‐related blurring and partial voluming of myocardium and buffer to non‐mono‐exponential diffusion signal behavior. This effect is also present elsewhere along the myocardial surfaces. The benefits of using DTV may be further enhanced in applications with greater microstructural heterogeneity such as myocardial infarction, and this is the subject of future investigation. Importantly, these findings demonstrate that the utility of DTV extends beyond DTI to more extensive *q*‐space sampling approaches, in which acceleration becomes even more critical.

Here, we applied DTV in the ex vivo heart. Although the technique would be applicable in vivo, additional issues need to be considered. For example, the use of single‐shot EPI may limit the application of compressed sensing due to the impracticalities of irregular k‐space sampling, and the super‐resolution approach may be more appropriate. The DTV algorithm also requires good alignment of the reference and target image. Therefore, it is important to design sequences that can be undersampled without changing the distortion profile relative to a fully sampled reference image. The algorithm assumes that edges are spatially co‐located in the reference and target image. As shown by Ehrhardt et al, this creates the potential to suppress real edges or create false edges in the target image if this assumption is violated. One way to reduce distortion, and simultaneously increase resolution, is the use of multishot fast spin‐echo sequences, including the twin navigator^35^ and split echo approaches.^36^ This could be combined with a weighted k‐space sampling scheme and T_2_ correction for compressed sensing in fast spin echo.^16^ However, there is the challenge of validating in vivo measurements due to the inherently lower imaging resolution and lack of a suitable high‐resolution ground truth. This limitation was observed in our reconstruction of DW data at 33 μm^3^ isotropic resolution. Although we show potential improvements, acquisition of such high‐resolution DW data may be impractical, due primarily to inadequate SNR. This difficulty is compounded in vivo due to motion and distortion. Although in vivo implementation will be challenging, we foresee that unconventional k‐space sampling schemes will provide opportunities to exploit the proposed DTV technique, and this is the subject of future work.

## 6 CONCLUSIONS

The DTV algorithm of Ehrhardt et al was introduced to address the problem of accelerating DWI. Two important purposes of DTV are to (1) improve the accuracy in quantification of data acquired with accelerated imaging methods, such as compressed sensing and super‐resolution, and (2) to enable acquisition of large data sets, such as multiple *q*‐shell diffusion MRI, not otherwise possible with full k‐space sampling. Although applied in the heart in this context, the algorithm is non‐application‐specific. The algorithm was used in the context of reconstructing DW data undersampled by up to 10×, as well as sharpening and removing ringing artifacts from images with 10× lower resolution than the reference image. One important feature of the algorithm is that it is contrast‐independent, using only on the location and orientation of edges in a reference image. Furthermore, it does not require any training data or impose any particular diffusion model on the data, but could potentially yield even better results when applied in combination with these types of reconstruction algorithms.

## CONFLICT OF INTEREST

A.D.M. and J.H.O. are co‐founders of and have an equity interest in Insilicomed, and A.D.M. has an equity interest in Vektor Medical. A.D.M. and J.H.O. serve on the scientific advisory board of Insilicomed, and A.D.M. as scientific advisor to both companies. Some of their research grants have been identified for conflict of interest management based on the overall scope of the project and its potential benefit to these companies. The authors are required to disclose this relationship in publications acknowledging the grant support; however, the research subject and findings reported in this study did not involve the companies in any way and have no specific relationship with the business activities or scientific interests of either company. The terms of this arrangement have been reviewed and approved by the University of California San Diego in accordance with its conflict of interest policies. The other authors have no conflicts of interest to declare.
